# A Central Board of Health for London

**Published:** 1901-09-14

**Authors:** 


### A Central Board of Health for London.

The epidemic of smallpox which has lately been going on in London seems to be coming to an end, although in view of the time of incubation taken by this disease it is too early as yet to feel sure that we are yet out of the wood. So far as it has gone the history of this outbreak has shown how effectually a small epidemic may be controlled when the appliances necessary for the purpose are kept in constant readiness, and those good people whose philosophy is expressed in the adage that "the proof of the pudding is in the eating," will no doubt say that we may justly plume ourselves on the satisfactory nature of our arrangements. Nevertheless, our arrangements partake largely of the ridiculous, and are such as certainly would never be allowed to continue if the health of London were handed over to the care of one single responsible body. Here we have London threatened with a great epidemic of a most dangerous, disgusting, and disfiguring disease. Where is the authority that steps in and takes control? There is no such authority. The County Council has nothing to do with it except so far as seeing that the local authorities maintain a certain standard of general sanitation. Then as to these local authorities. They can, of course, set their sanitary inspectors to trace out the cases and investigate the sources of the infection, and this they have done with great perseverance and success. But when they have gone so far, and have adopted certain measures for disinfection, they have come to the end of their tether. They cannot remove or isolate the cases, for they have no hospitals to remove them to. Directly it becomes a matter of isolation and hospital treatment, the local sanitary authorities have to stand aside and leave everything to an independent Board on which neither they nor the central health authority, the County Council, have a single representative, and over the doings of which they have not the slightest control. We have not a word to say against the Metropolitan Asylums Board. The work it has done in providing hospitals, in removing the infected by means of its splendid ambulance service, in caring for its patients, and latterly in encouraging scientific work within its hospitals, has been in every way excellent, so excellent as to have excited the admiration of visitors interested in medicine and hygiene from all parts of the world. But it is an independent body, it forms no integral part of, nor is it under any legal obligation to, those local sanitary authorities to which the care of the health of London has by law been handed over, and by its existence these local bodies are practically debarred from setting up any machinery of their own for dealing with infectious diseases.

The principal means at our disposal for controlling an outbreak of smallpox are vaccination, notification, and isolation. Notification, as in every other town, is made direct to the district medical officers of health. But the co-ordinating notification authority for the whole of London is not, as one would expect it to be, the County Council, but strangely enough the Metropolitan Asylums Board.

Then about vaccination. This is our great protection against smallpox. It is a purely sanitary measure, a detail of preventive medicine. Yet it is taken entirely out of the hands of the sanitary authorities, and given over to—of all people in the world—the poor-law guardians. These good people may or may not turn stupid about it, but in any case they have no direct connection whatever with the machinery for the control of an epidemic. As a last resource then, what about the Metropolitan Asylums Board? It is on to this board that every other body throws the work, and so long as the outbreak is a little one, well within its powers, all goes on satisfactorily. But it has no responsibility in the matter. As long as it has beds at liberty it fetches in and receives the cases, but when its accommodation is exhausted, as we have seen happen again and again with diphtheria, its duty is done and it thinks no more of declining to take in cases than a theatre manager does of saying "House Full." Then London must simmer in its own juice until a vacancy occurs. The whole arrangement is absurd.

Again it is well known that a large number of medical men, having never or hardly ever seen a case of smallpox, find considerable difficulty in diagnosing it in its early stages. If any one single sanitary authority had to deal with the epidemic, surely it would employ a few experts to visit and investigate all doubtful cases, as is done in other countries, and as has been already arranged to be done in regard to plague. But that is far too simple a proceeding. We have the experts, and we pay for them through the Asylums Board; but it must be understood that the duty of this Board is not to control an epidemic, but to take care that no one not suffering from smallpox gets into its hospitals. That is all its interest in the matter; so it establishes its experts at the wharf whence its ambulance steamers start! Instead of the doubtful cases being visited and examined before removal, they have to be dragged out of bed and trundled all the way down to Rotherhithe to be told, perhaps, that after all they have only got chicken-pox! London is in many ways a charming place, but for muddle it is absolutely incomparable. There should be one Central Board of Health for the whole of the Metropolis.

